# A Novel Approach of Feature Space Reconstruction with Three-Way Decisions for Long-Tailed Text Classification

**DOI:** 10.1155/2022/3183469

**Published:** 2022-04-16

**Authors:** Xin Li, Lianting Hu, Peixin Lu, Tianhui Huang, Wei Yang, Quan Lu, Huiying Liang, Long Lu

**Affiliations:** ^1^School of Information Management, Wuhan University, Wuhan, China; ^2^Medical Big Data Center, Guangdong Provincial People's Hospital, Guangdong Academy of Medical Sciences, Guangdong, China; ^3^Institute of Pediatrics, Guangzhou Women and Children's Medical Center, Guangzhou Medical University, Guangzhou, China; ^4^Guangdong Cardiovascular Institute, Guangzhou, Guangdong, China; ^5^Wuhan National Laboratory for Optoelectronics, Huazhong University of Science and Technology, Wuhan, China

## Abstract

Text classification is widely studied by researchers in the natural language processing field. However, real-world text data often follow a long-tailed distribution as the frequency of each class is typically different. The performance of current mainstream learning algorithms in text classification suffers when the training data are highly imbalanced. The problem can get worse when the categories with fewer data are severely undersampled to the extent that the variation within each category is not fully captured by the given data. At present, there are a few studies on long-tailed text classification which put forward effective solutions. Encouraged by the progress of handling long-tailed data in the field of image, we try to integrate effective ideas into the field of long-tailed text classification and prove the effectiveness. In this paper, we come up with a novel approach of feature space reconstruction with the help of three-way decisions (3WDs) for long-tailed text classification. In detail, we verify the rationality of using a 3WD model for feature selection in long-tailed text data classification, propose a new feature space reconstruction method for long-tailed text data for the first time, and demonstrate how to effectively generate new samples for tail classes in reconstructed feature space. By adding new samples, we enrich the representing information of tail classes, to improve the classification results of long-tailed text classification. After some comparative experiments, we have verified that our model is an effective strategy to improve the performance of long-tailed text classification.

## 1. Introduction

**Figure 1:**
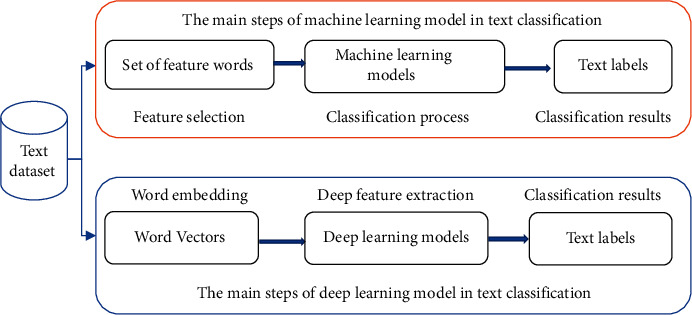
The main steps based on machine learning and deep learning models in text classification.

Due to the rapid development of Internet technology and information construction, it becomes easier to obtain valuable text data to study and analyze problems in certain fields. As a consequence, text classification is widely studied by researchers in the natural language processing field [1–3], and many applications based on text classification technology have been developed, such as news filtering and organization [4], e-mail classification and spam filtering [5], web page mining [6], and medical documents' classification [3]. Text classification is a task that assigns textual documents to predefined classes based on the knowledge extracted from their content [7]. A wide variety of techniques has been designed for text classification, which is mainly based on machine learning models, such as Naive Bayes (NB) [8], Decision Tree (DT) [9], Support Vector Machines (SVMs) [10], and deep learning models, such as convolutional neural networks (CNNs) [11, 12], recurrent neural network (RNN) [13], bidirectional long short-term memory (BI-LSTM) [14, 15], and transformer models [16]. Calculating text representation, training classification models, and predicting class labels for class-unknown documents are the main steps of text classification [17]. The main steps based on these models in text classification are shown in Figure 1.

As we all know, sufficient data are the premise of superior performances of these artificial intelligence learning methods; especially, deep learning models are developed to train massive data initially [18, 19]. But real-world text data often follow a long-tailed distribution as the frequency of each class is typically different, such as news topic classification, clinical name entities recognition, and disease diagnosis for electronic medical records [20–22]. It means that a dataset can have a large number of under-represented classes (tail classes) and a few classes with more than sufficient data (head classes). The performance of supervised learning algorithms in the process of text classification suffers when the training data are highly imbalanced [23, 24]. The problem can get worse when the categories with a fewer data are severely undersampled to the extent that the variation within each category is not fully captured by the given data.

At present, few studies have studied the problem of long-tailed text classification and put forward effective solutions. Some methods have been proposed to handle the problem of imbalanced datasets in text classification [25–29]. Finding and fine tuning the network model of classifiers suitable for specific data sets is one of these methods, such as [25] describing the proposed approach for text classification in an unbalanced data environment based on an implementing individual LSTM neural network. Data augmentation is also a frequently used way in scenarios with few samples or unbalanced categories [26–29], such as back translation [26], which has become an effective way of data augmentation; for example, researchers translate some English text into Chinese and then translate them back into English, they can get various new training data, and the size of the dataset is doubled. Oversampling [27] and undersampling [28] are both common methods to deal with unbalanced datasets. But those methods which repeatedly take samples only consider the processing at the data level, so the generated data are still redundant and invalid in the feature space, and the improvement of classification effect is not obvious. The method of finding and fine tuning appropriate classifiers in specific data sets does not have universal applicability. At present, research studies on real-world long-tailed data mainly focus on the field of image classification [30–34].

Those techniques for learning long-tailed distributions generally fall into three groups: resampling [30], reweighting and cost-sensitive learning [31, 32], and feature manipulation [33, 34]. Especially, methods based on feature space augmentation of long-tailed image data have effectively improved the classification effect recently [33, 34].

**Figure 2:**
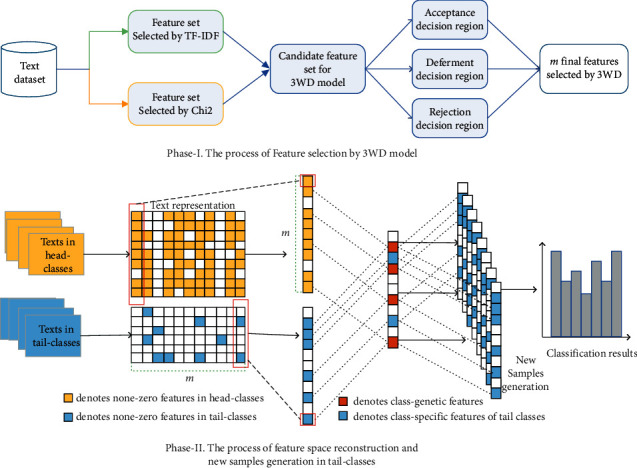
The proposed model architecture in our paper.

Encouraged by the progress of handling long-tailed data in the field of image [30–34], considering texts carry a lot of semantic information, analysis is more complex than image data, we try to integrate effective ideas into the field of long-tailed text classification, and prove the effectiveness. In this paper, we come up with a novel approach of feature space reconstruction with 3WD for long-tailed text classification in Figure 2. In detail, with the help of 3WD model, the main innovations of this paper can be summarized into three aspects: (1) We verify the effectiveness of using a 3WD model for feature selection in long-tailed text data classification. (2) We propose a new feature space reconstruction method for long-tailed text classification and demonstrate how to effectively generate new samples for tail classes in reconstructed feature space. By adding new samples, we enrich the representing information of tail classes, to improve the classification results of long-tailed text classification. (3) After some comparative experiments, we have verified that our model is an effective strategy to improve the performance of long-tailed text classification, which can effectively improve the accuracy values of tail classes. The rest of paper is organized as follows: Section 2 introduces the novel approach of feature space reconstruction with 3WD for long-tailed text classification related works. In Section 3, we design some experiments and provide the corresponding results in detail. Finally, we draw some conclusions and some possible future works in Section 4.

## 2. Methods

In this section, we propose a novel approach of feature space reconstruction with 3WD for long-tailed text classification. As mentioned above, calculating text representations, training classification models, and predicting class labels for class-unknown documents are the main steps of text classification. Firstly, with the help of 3WD, two evaluation functions are used to generate the optimal set of features, which can effectively reduce the number of feature words, make the extracted feature words more representative of categories, and text representations can be calculated accordingly. Then, because texts containing sufficient information in tail-class data only account for a small part, some information will be lost in the process of text representations, and it is easy to find that the representation vectors of tail classes are very sparse. Thus, we augment the data in tail classes by reconstructing the feature space with the features learned from the head classes with ample samples. In particular, we decompose the features of each class into class-generic features and class-specific features. Novel samples of tail classes are then generated randomly by fusing the class-specific features from the tail classes with the class-generic features from head classes. Finally, the new samples are added to existing data to train the classification models and predict class labels to verify the effect of our method.

### 2.1. Feature Selection by 3WD

Considering the decision risk and uncertainty, the 3WD method was initially proposed by Yao [35] based on rough sets and Bayesian decision procedure. The 3WD method which has attracted extensive attention in various fields provides a new tool for text classification [36–38]. The core idea of the 3WD method is to divide the universe into three disjoint regions, including an acceptance decision region, a deferment decision region, and a rejection decision region. If the decision maker has enough information, he (or she) can quickly make a decision, i.e., acceptance and rejection. Otherwise, the decision maker can choose to postpone the decision.

Let the dataset of long-tailed texts denotes as *X*={*X*_Head_,*X*_Tail_}={*x*_1_, *x*_2_,…, *x*_*i*_,…, *x*_*n*_}, where *X*_Head_ is the set of the classes with ample samples (head classes), *X*_Tail_ is the set of the samples of under-represented classes (tail classes), and the total amount of all texts in *X* is *n*. *C*={*C*_Head_, *C*_Tail_}={*C*_1_, *C*_2_,…, *C*_*k*_,…, *C*_*K*_}, *C*_*k*_ denotes the kth class of *X*, and there are *K* classes in *X*. Firstly, we need to preprocess the long-tailed samples and do word segmentation for Chinese texts. *V*={*v*_1_, *v*_2_,…, *v*_*j*_,…, *v*_*N*_} be the vocabulary mined from *X*.

The 3WD model used in this paper uses the TF-IDF algorithm [39] and Chi2 Statistics [40] as double-decision functions to select features in the long-tailed dataset, which can consider the frequency and distribution of vocabularies, as well as label-related information. *M*_*μ*_ is defined as the feature set selected by decision function *μ*, and *M*_*ω*_ is defined as the feature set selected by decision function *ω*. For ∀*v*_*j*_ ∈ *V*:

(1) $$\begin{array}{c} \mu \left(v_{j}\right)=\text{ TF}\left(v_{j},x_{i}\right)\;\times \text{ IDF}=\;\frac{n_{j,i}}{\sum_{s}n_{s,i}}\times lg\frac{\left|n\right|+1}{\left|\left\{i:v_{j}\in x_{i}\right\}\right|+1}\;. \end{array}$$

Here, *n*_*i*,*j*_ denotes the number of times vocabulary *v*_*j*_ appeared in document *x*_*i*_, ∑_*k*_*n*_*k*,*i*_ is the total times of all vocabularies that appeared in document *x*_*i*_, and the total amount of all texts in *X* is |*n*|. |{*i* : *v*_*j*_ ∈ *x*_*i*_}| is the total number of documents with *v*_*j*_. Adding 1 to prevent *v*_*j*_ is not in *X*, resulting in a calculation error caused by zero denominators.

(2) $$\begin{array}{c} \omega \left(v_{j}\right)=x^{2}\left(v_{j},C_{k}\right)=\frac{n{\left(A_{jk}D_{jk}-C_{jk}B_{jk}\right)}^{2}}{\left(A_{jk}+C_{jk}\right)\left(B_{jk}+D_{jk}\right)\left(A_{jk}+B_{jk}\right)\left(D_{jk}+C_{jk}\right)}, \end{array}$$

where *A*_*jk*_ is the number of documents that contain *v*_*j*_ and belong to *C*_*k*_, *B*_*jk*_ is the number of documents that contain *v*_*j*_ but do not belong to *C*_*k*_, *C*_*jk*_ is the number of documents that do not contain *v*_*j*_ but belong to *C*_*k*_, and *D*_*jk*_ is the number of documents that do not contain *v*_*j*_ and not belong to *C*_*k*_.

The feature sets *M*_*μ*_ and *M*_*ω*_ of the longed-tailed text data are generated by two evaluation functions, we map the feature words existing in both feature sets to the positive field POS(*μ*, *ω*) of the 3WD model. The feature words that only exist in one feature set *M*_*μ*_ or *M*_*ω*_ are added to the boundary domain BND(*μ*, *ω*) of the 3WD model, further processing is carried out with these features according to the classification results to decide to retain them or not, and the feature words with no appearance in feature sets are added to the negative domain NEG(*μ*, *ω*). 3WD rules can be constructed through three fields: the positive field POS(*μ*, *ω*)  corresponds to acceptance, the negative field NEG(*μ*, *ω*)  corresponds to rejection, and the boundary field BND(*μ*, *ω*) corresponds to non commitment. Then, ∀*v*_*j*_ ∈ *V*:

(3) $$\begin{array}{c} \left\{\begin{array}{c} v_{j}\in \text{POS}\left(\mu ,\omega \right),\;if\;v_{j}\in \;M_{\mu }\;\mathrm{and}\;v_{j}\in \;M_{\omega },\\ v_{j}\in \text{BND}\left(\mu ,\omega \right),\text{ if }\left(v_{j}\in \;M_{\mu }\text{ but }v_{j}\notin \;M_{\omega }\right)\;\mathrm{or}\;\left(v_{j}\notin \;M_{\mu }\;\mathrm{but}\;v_{j}\in \;M_{\omega }\right),\\ v_{j}\in \text{NEG}\left(\mu ,\omega \right)\text{, if }v_{j}\notin \;M_{\mu }\;\mathrm{and}\;v_{j}\notin \;M_{\omega }. \end{array}\right. \end{array}$$

and NEG(*μ*, *ω*)=(POS(*μ*, *ω*)∪BND(*μ*, *ω*))^*C*^.

The final feature set *T*={*t*_1_, *t*_2_,…, *t*_*m*_} is generated accordingly, which is a collection of *m* feature words used for classification. For the effectiveness of this method for feature selection in the process of long-tailed text data classification, we evaluate in Section 3.2 of the paper.

### 2.2. Feature Space Reconstruction and Novel Sample Generation for Tail Classes

In Section 2.1, we obtain the final feature set *T*={*t*_1_, *t*_2_,…, *t*_*m*_} with *m* optimal feature words; thus, document *x*_*i*_  can be represented as a vector of features *F*_*i*_={*f*_1_, *f*_2_,…, *f*_*m*_} , and the collection of long-tailed text data *X* can be represented as equation:

(4) $$\begin{array}{c} M=\;\left[\begin{array}{ccc} \begin{array}{cc} f_{11} & f_{12}\\ f_{21} & f_{22} \end{array} & \cdots & \begin{array}{c} f_{1m}\\ f_{2m} \end{array}\\ \vdots & \ddots & \vdots \\ \begin{array}{cc} f_{n1} & f_{n2} \end{array} & \cdots & f_{\text{nm}} \end{array}\right]\;, \end{array}$$

where element *f*_*ij*_  represents the binary weight of *t*_*j*_ from *F*_*i*_. If *t*_*j*_ appears in *x*_*i*_, *f*_*ij*_=1, and otherwise *f*_*ij*_=0.

Thus, for a given class *C*_*k*_, the matrix of *C*_*k*_ can be expressed as equation:

(5) $$\begin{array}{c} M_{C_{k}}=\;\left[\begin{array}{ccc} \begin{array}{cc} f_{11} & f_{12}\\ f_{21} & f_{22} \end{array} & \cdots & \begin{array}{c} f_{1m}\\ f_{2m} \end{array}\\ \vdots & \ddots & \vdots \\ \begin{array}{cc} f_{S1} & f_{S2} \end{array} & \cdots & f_{Sm} \end{array}\right]. \end{array}$$

Then, we can get the class feature vector of *C*_*k*_ which is denoted as *F*_*C*_*k*__={*f*_1_^*k*^, *f*_2_^*k*^,…, *f*_*m*_^*k*^}, where *f*_*i*_^*k*^=sgn(∑_*S*_*f*_*si*_), sgn(*x*)=1 for *x* > 0,  sgn(*x*)=0  for *x*=0. *F*_*C*_*k*__ represents the binary weight of feature words in all documents of class *C*_*k*_, namely, if *t*_*j*_ appears in *x*_*i*_, *f*_*ij*_=1, otherwise *f*_*ij*_=0.

By comparing with head classes, we find that the class feature vectors of tail classes are extremely sparse, which contain small numbers of nonzero eigenvalues. This is because texts containing sufficient information in tail-class data only account for a small part, and some information is lost in the process of text representations, which leads to poor classification results. The data in the head class are sufficient. It seems natural to use the knowledge learned from the head class to help recover the missing information in the tail class.

In [34], authors use deep convolution neural networks to augment the feature space of long-tailed image data. Through image feature sampling, the class features of these images are divided into class-generic and class-specific features. It is proved that the class general features from the head class are regarded as transferable knowledge for the feature space expansion of the tail class, and the effectiveness of this idea is proved in the image filed.

In this paper, we explore the effectiveness of feature reconstruction in long-tailed text data classification. For a given head class and a tail class, *F*^head^={*f*_1_^*h*^, *f*_2_^*h*^,…, *f*_*m*_^*h*^} and *F*^tail^={*f*_1_^*t*^, *f*_2_^*t*^,…, *f*_*m*_^*t*^} are class feature vectors of them correspondingly, and *m* is the total number of feature words. By analyzing their class feature sets, we map the feature words existing in both class feature sets to the class-generic feature set *M*^*G*^, and the feature words only existing in tail-class feature set to tail class-generic feature set *M*^*TS*^, thus for ∀*t*_*i*_ ∈ *T*:

(6) $$\begin{array}{c} \left\{\begin{array}{c} t_{i}\in M^{G},\;\mathrm{if}\;f_{i}^{h}*f_{i}^{t}=1,\\ t_{i}\in M^{TS},\mathrm{if}\;f_{i}^{h}*f_{i}^{t}=0\text{ and sgn}\left(f_{i}^{t}-f_{i}^{h}\right)=1. \end{array}\right. \end{array}$$

Using the class-generic feature set *M*^*G*^ and *t* tail class-generic feature set *M*^*TS*^, we reconstruct the feature space of tail-class documents. The class-specific features from the class are then combined with the class-generic features from the Na classes linearly. A random combination ratio is generated to guide the fusion by randomly drawing class-generic and class-specific feature vectors to form an augmented sample for the tail class.

### 2.3. Long-Tailed Text Classification

In Section 2.2, novel samples of tail classes are generated by fusing the class-specific features from the tail classes with the class-generic features from classes with ample data. Finally, the new samples are added to existing data to train the classification models and predict class labels to verify the effect of our method. In our paper, we use XGBoost as our classifier which is a method of gradient boosting decision tree.

We evaluate the classification performance of the proposed method in our paper and compare it with other existing machine learning and deep learning methods. For each class *C*_*k*_, we use accuracy precision, recall, and F1-measure as experiment metrics defined in Table 1.


(7) $$\begin{array}{c} \text{Accuracy}\left(C_{k}\right)=\frac{\text{TP}\left(C_{k}\right)+\text{TN}\left(C_{k}\right)}{\text{TP}\left(C_{k}\right)+\text{TN}\left(C_{k}\right)+\text{FP}\left(C_{k}\right)+\text{FN}\left(C_{k}\right)},\\ \text{Precision}\left(C_{k}\right)=\frac{\text{TP}\left(C_{k}\right)}{\text{TP}\left(C_{k}\right)+\text{FP}\left(C_{k}\right)}\;,\\ \text{Recall}\left(C_{k}\right)=\frac{\text{TP}\left(C_{k}\right)}{\text{TP}\left(C_{k}\right)+\text{FN}\left(C_{k}\right)},\\ \text{F}{}_{1}\;\left(C_{k}\right)=\frac{2*\text{Precision}\left(C_{k}\right)*\text{Recall}\left(C_{k}\right)}{\text{Precision}\left(C_{k}\right)+\text{Recall}\left(C_{k}\right)}. \end{array}$$

In multiclass classification problems, the overall performance can be measured by averaging the evaluation methods. Microaverage and macroaverage are used widely for this purpose. In this study, in cases of evaluating long-tailed distribution, it is better to use macroaverage scores than microaverage scores since the data size of categories is not considered in the microaverage calculation. Taking the definition of F1-value as an example, the definitions of macro-F1 and micro-F1 are shown in equations as follows:

(8) $$\begin{array}{c} \text{Macro}-\text{F}1=\frac{1}{K}\overset{K}{\underset{i=1}{\sum }}F_{1}\left(C_{k}\right),\\ \text{Micro}-\text{F}1=\frac{2*\sum_{i=1}^{K}\text{TP}\left(C_{k}\right)}{2*\sum_{i=1}^{K}\text{TP}\left(C_{k}\right)+\sum_{i=1}^{K}\text{FP}\left(C_{k}\right)+\sum_{i=1}^{K}\text{FN}\left(C_{k}\right)}. \end{array}$$

## 3. Experiments

### 3.1. Dataset

**Figure 3:**
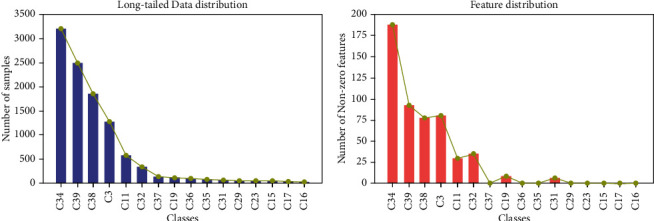
(a) The distribution of our long-tailed text dataset; (b) the distribution of features.

In this paper, we use Fudan University corpus as a long-tailed text classification corpus. The Fudan University TC corpus is from the Chinese NLP group in the Department of Computer Information and Technology, Fudan University of China. We randomly selected some category data to simulate the distribution of real-world long-tailed datasets. The numbers and distribution of our dataset can be seen in Table 2 and Figure 3. There are 4 head classes and 12 tail classes. The ratio of training set to test set is 7 : 3.

In this study, we carried out 3 experiments on the dataset. All experiments were implemented on a 64 bit MAC computer with 8 GB internal storage. The experimental code was written in Python language using Scikit-learn (sk-learn) and TensorFlow. Sk-learn and TensorFlow are commonly used third-party modules in machine learning and deep learning which encapsulate many commonly used machine learning and deep learning algorithms, such as SVM, XGBoost, RNN, CNN, and others. In preprocessing, all documents were segmented into words by the opensource tool Jieba, and stop words were removed in this process.

By the final feature set obtained by the 3WD model, we can calculate the text representations of our long-tailed text dataset described in Section 2.1. And the number of nonzero features of each class is obtained, as shown in Figure 3(b). It can be seen that the tail classes contain less information, and some information is lost in the process of text representations; it is easy to find that the representation vectors of tail class are very sparse.

### 3.2. The Effectiveness of Feature Selection Using 3WD

In this paper, we use the 3WD model to generate the final feature set *T*={*t*_1_, *t*_2_,…, *t*_*m*_} of long-tailed text data, which is a collection of *m* feature words used for classification. In this section, we use word frequency, CHI2 [40], and TF-IDF [39] as comparative methods for feature selection, to test the effectiveness of the 3WD model in our dataset. For all methods mentioned above, we select the top 2,000 feature words and use SVM as a classification model. The corresponding results are shown in Table 2.

From the results of Table 3, we can see that the 3WD algorithm has improved the long-tailed text classification performance in the scores of accuracy, precision, recall, and F1-value than other methods. It is about 10～20% improvement than other methods on average. From the average point of view, 3WD model's overall performance is better than other methods.

### 3.3. The Effectiveness of Feature Reconstruction of Tail Class

In Section 2.2, we augment the tail-class data by reconstructing the feature space with the features learned from the head classes with ample samples. In particular, we decompose the features of each class into class-generic features and class-specific features. Novel samples of tail classes are then generated randomly by fusing the class-specific features from the tail classes with the class-generic features from head classes. In this section, the new samples are added to existing data to train the classification models and predict class labels to verify the effect of our method. According to the number of samples generated, it is divided into 6 levels, namely, 0%, 10%, 20%, 30%, 40%, 50%, and 60%, which represent the degrees of sample generation.

**Figure 4:**
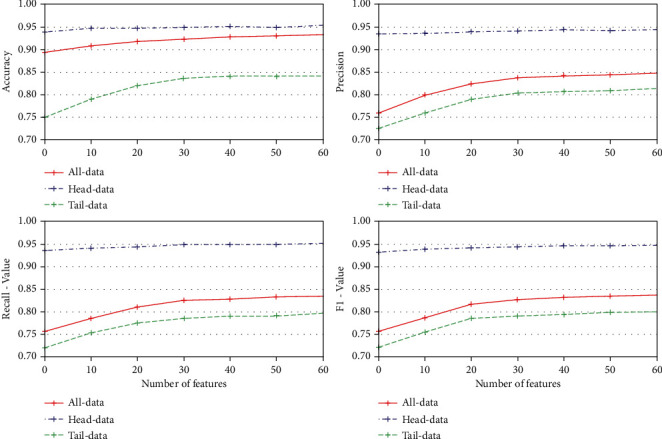
The performance results of our proposed model with different generation degrees.

In the first stage of our experiment, we use the 3WD algorithm as a feature selection method to select 2,000 effective feature words; the introduction and distribution of our long-tailed text dataset can be seen in Section 3.1. From the results of Figure 4, we can see that when we add new samples of tail-class data by reconstructing the feature space by learning information from head-classes based on the original samples, the classification accuracy is significantly improved from 0 to 30% generation degree; the experiment shows that the classification results are not significantly improved after 30–40% generation degree. Especially, the accurate score of tail-class data has improved from 0.752 to 0.84 when the sample generation degree increased to 30%, from 30% degree to 60% degree, and the accuracy is not improved. The precision score of tail-class data has improved from 0.725 to 0.813, the recall score of tail-class data has improved from 0.72 to 0.797, the F1-value score of tail-class data has improved from 0.722 to 0.801 when the sample generation degree increased to 30%. It can also be seen from Figure 4 that the classification results of tail-class data have improved without reducing the accuracy of all data and head-class data.

### 3.4. Comparing with Mainstream Methods

In our experiments, we use XGBoost as our classifier and compare it with four baseline training methods: TF-IDF with Linear SVM, CNN, RNN, and Bi-LSTM. XGBoost is a method of gradient boosting decision tree. It strives to maximize speed and efficiency, so it is called *X* (extreme) gboosted. Parameter settings for XGBoost in our experiments: max_depth:6, eta:0.5, objective: multi: SoftMax, and num_class:16.

Convolutional neural network (CNN) is a variant of multilayer perceptron (MLP), which essentially is a multilayer perceptron. The key to its success lies in its way of local connection and sharing weights. On the one hand, it reduces the number of weights, which makes the network easy to optimize and reduces the risk of overfitting. Parameter settings for CNN in our experiments: embedding_dim = 100, seq_length = 600, num_classes = 16, num_filters = 256, kernel_size = 5, hidden_dim = 128, dropout_keep_prob = 0.5, learning_rate = 1e-3, and batch_size = 64.

RNN (recurrent neural network) is a kind of a neural network used to process sequence data. The biggest difference of RNN is that it also establishes weight connections between neurons and between layer to capture the information at previous time points. Parameter settings for RNN in our experiments: embedding_dim = 100, seq_length = 600, num_classes = 16, num_layers = 3, hidden_dim = 128, rnn = “gru,” dropout_keep_prob = 0.8, learning_rate = 1e-3, and batch_size = 128.

Bi-LSTM is the abbreviation of bidirectional long short term memory, which is a combination of forwarding LSTM and backwarding LSTM. Both are often used to model context information in natural language processing tasks. LSTM is a kind of RNN that is very suitable for modeling time-series data, such as text data. Parameter settings refer to RNN.

**Figure 5:**
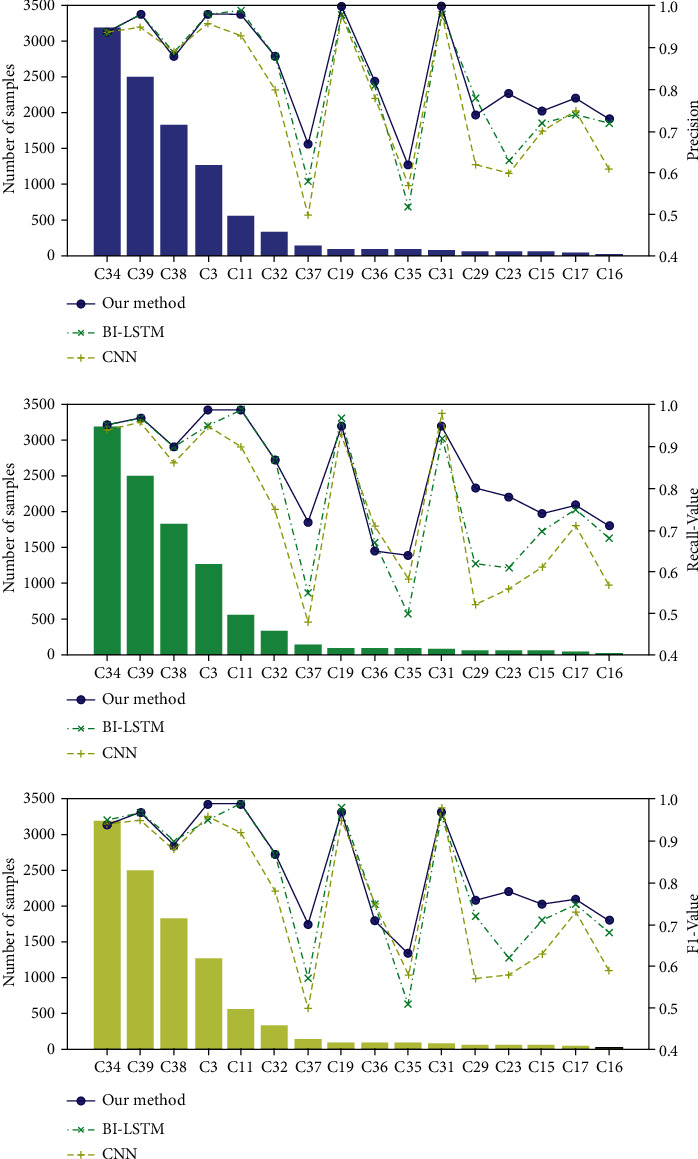
The performance results of our proposed model and deep learning methods.

In this section, the results of accuracy, macroprecision, macrorecall, and macro-F1 score in our experiment are shown in Table 4 and Figure 5. It is easy to see that the proposed method in our paper outperformed baseline training methods in all cases, especially in tail classes. The macro-F1 score obtained by our method in tail classes is 80.1%, which is 5∼10% higher than that obtained by deep learning models and 20% higher than that obtained by TF-IDF. Meanwhile, other results reflect similar improvements to our method.

## 4. Discussion

Long tail text classification is a problem that needs to be solved in the real world. The performance of current mainstream learning algorithms in text classification suffers when the training data are highly imbalanced. In this paper, a novel approach of feature space reconstruction with the help of 3WD for long-tailed text classification is proposed to greatly reduce the impact from the long-tailed distribution of datasets. In detail, we use Fudan University corpus as a long-tailed text classification corpus with 4 head classes and 12 tail classes, and verify the effectiveness of using the 3WD model for feature selection in long-tailed text data. Then, we propose a new feature space reconstruction method for long-tailed text data and demonstrate how to effectively generate new samples for tail classes in reconstructed feature space. By adding new samples, we enrich the representing information of tail classes, to improve the classification results of long-tailed text classification. After performing some comparative experiments, we have verified that our model is an effective strategy to improve the performance of long-tailed text classification with the comparison with mainly used deep learning methods.

As mentioned above, real-world text data often follow a long-tailed distribution as the frequency of each class is typically different. For example, the classification of diseases in the auxiliary diagnosis model based on electronic medical records usually presents a long tail distribution; the distribution of different types of entities extracted from biomedical texts often shows long-tailed state. Rather than using back translation for data augmentation, this paper verifies the effectiveness of feature space reconstruction for long-tailed data classification. In the next research, we will apply the method in this paper to the construction of an auxiliary diagnosis model to verify the effectiveness of our method and whether it can improve the accuracy of disease diagnosis.

## Figures and Tables

**Table 1:** Meanings of TP, TN, FP, and FN.

Sample *x*_*i*_ in the corpus *X*	Result in *C*_*k*_	Not a result in *C*_*k*_
Belongs to *C*_*k*_	TP(*C*_*k*_)	FP(*C*_*k*_)
Does not belong to *C*_*k*_	FN(*C*_*k*_)	TN(*C*_*k*_)

**Table 2:** The numbers of the long-tailed text dataset used in our paper.

No.	Class	Class label	Samples
1	Head	C34-Economy	3201
2	Head	C39-Sports	2507
3	Head	C38-Politics	1854
4	Head	C3-Art	1282
5	Tail	C11-Space	582
6	Tail	C32-Agriculture	348
7	Tail	C37-Military	150
8	Tail	C19-Computer	120
9	Tail	C36-Medical	104
10	Tail	C35-Law	103
11	Tail	C31-Environment	89
12	Tail	C29-Transport	67
13	Tail	C23-Mine	67
14	Tail	C15-Energy	65
15	Tail	C17-Communication	55
16	Tail	C16-Electronics	32

**Table 3:** The performance results of 3WD models and baseline methods for feature selection.

Method	Accuracy	Macroprecision	Macrorecall	Macro-F1 score
Word frequency	0.56	0.487	0.518	0.51
Chi2	0.70	0.577	0.591	0.58
TF-IDF	0.79	0.638	0.627	0.631
3WD (our paper)	0.87	0.697	0.637	0.656

**Table 4:** The performance results of our proposed model and baseline methods.

Method	Datatype	Accuracy	Macroprecision	Macrorecall	Macro-F1 score
TF-IDF	All data	0.79	0.65	0.635	0.64
	Head class	0.872	0.854	0.857	0.857
	Tail class	0.635	0.597	0.581	0.585

CNN	All data	0.915	0.765	0.732	0.747
	Head class	0.943	0.937	0.928	0.933
	Tail class	0.747	0.731	0.692	0.709

RNN	All data	0.905	0.783	0.767	0.775
	Head class	0.932	0.927	0.916	0.918
	Tail class	0.782	0.759	0.728	0.742

BI-LSTM	All data	0.927	0.82	0.796	0.81
	Head class	0.954	**0.947**	0.94	0.943
	Tail class	0.821	0.778	0.747	0.752

Our method	All data	**0.933**	**0.842**	**0.825**	**0.832**
	Head class	0.953	0.945	**0.951**	**0.948**
	Tail class	**0.841**	**0.813**	**0.797**	**0.801**

Bold values means the best values in accuracy, macroprecision, macrorecall, and macro-F1 score.

## Data Availability

This study uses the Fudan University TC corpus which is from the Chinese NLP group in Department of Computer Information and Technology, Fudan University of China. All data are public and available online.
